# Patient Experiences Using a Self-Monitoring App in Eating Disorder Treatment: Qualitative Study

**DOI:** 10.2196/10253

**Published:** 2018-06-22

**Authors:** Pil Lindgreen, Kirsten Lomborg, Loa Clausen

**Affiliations:** ^1^ Research Unit Center for Child and Adolescent Psychiatry Aarhus University Hospital Risskov Denmark; ^2^ Department of Clinical Medicine Faculty of Health Aarhus University Aarhus Denmark; ^3^ Department of Public Health Faculty of Health Aarhus University Aarhus Denmark; ^4^ Department of Psychology Behavioral and Social Sciences Aarhus University Aarhus Denmark

**Keywords:** feeding and eating disorders, anorexia nervosa, bulimia nervosa, mental health, psychiatry, mHealth, mobile applications, self-monitoring, blended treatment, eating disorder treatment

## Abstract

**Background:**

The Recovery Record smartphone app is a self-monitoring tool for individuals recovering from an eating disorder. Unlike traditional pen-and-paper meal diaries, which are often used in eating disorder treatment, the app holds novel features, such as meal reminders, affirmations, and patient-clinician in-app linkage, the latter allowing for clinicians to continuously monitor patients' app data.

**Objective:**

To explore patients' experiences with using Recovery Record as part of outpatient eating disorder treatment.

**Methods:**

A total of 41 patients from a Danish eating disorder treatment facility were included in the study. All 41 patients participated in participant observations of individual or group treatment sessions, and 26 were interviewed about their experiences with using the app in treatment. The data material was generated and analyzed concurrently, applying the inductive methodology of Interpretive Description.

**Results:**

The patients' experiences with Recovery Record depended on its app features, the impact of these features on patients, and their specific app usage. This patient-app interaction affected and was affected by changeable contexts making patients' experiences dynamic. The patient-app interaction affected patients' placement of specific Recovery Record app features along a continuum from supportive to obstructive of individual everyday life activities including the eating disorder treatment. As an example, some patients found it supportive being notified by their clinician when their logs had been monitored as it gave them a sense of relatedness. Contrarily, other patients felt under surveillance, which was obstructive, as it made them feel uneasy or even dismissing the app.

**Conclusions:**

Some patients experienced the app and its features as mostly supportive of their everyday life and the eating disorder treatment, while others experienced it primarily as obstructive. When applying apps in eating disorder treatment, we therefore recommend that patients and clinicians collaborate to determine how the app in question best fits the capacities, preferences, and treatment needs of the individual patient. Thus, we encourage patients and clinicians to discuss how specific features of the applied app affect the individual patient to increase the use of supportive features, while limiting the use of obstructive ones.

## Introduction

### Blended Treatment in Health Care Settings

Since the launch of mobile phones in 2007, the development of mobile phone apps has rapidly increased within health care settings; by 2017, more than 325,000 health-related apps were available to the large population of mobile phone users worldwide [1]. Correspondingly, “blended” treatment, namely, the mixture of digital tools and traditional face-to-face treatment, is becoming more common, although highly underresearched [2]. In many countries, digitized health care is encouraged politically because it is expected to bring about several benefits, such as a wider geographical outreach and reduced costs [3,4]. Additionally, several digital health tools aim at engaging patients in their treatment by performing self-monitoring activities, which is often helped by in-app nudging features [5-7].

### Eating Disorders and Self-Monitoring

Self-monitoring apps have been developed for several mental disorders, including eating disorders (EDs) [8]. EDs can have severe physiological and psychosocial consequences, for example, osteoporosis, infertility, depression, and social isolation [9,10]. The main EDs are anorexia nervosa, bulimia nervosa, and binge ED (BED). Anorexia nervosa entails self-inflicted underweight due to restrictive dieting, whereas bulimia nervosa involves episodes of binge eating. In both these EDs, different weight loss measures are applied, for example, excessive exercising and fasting in the restrictive subtype or vomiting and the use of laxatives and diuretics in the purging subtype. BED also includes binge eating but no regular compensatory weight loss behaviors [11]. In worst cases, EDs can be lethal [12], and standard mortality ratios are elevated, especially for anorexia nervosa [13,14]. Thus, effective ED treatment is crucial, although only 40%-70% fully recover, relapse is common [10], and treatment dropout rates are high, ranging from 29% to 73% in outpatient settings [15]. Normalizing patients’ eating patterns and weight is prioritized, especially in the initial treatment phases [16]; often, cognitive-behavioral therapy (CBT) is applied because it has been found to be effective for this purpose [17-19]. CBT also aims at patients gaining an understanding of what triggers and relieves their ED symptoms [20]. For these purposes, CBT in ED treatment employs self-monitoring activities where patients register information on their meals and their emotions, behavior, and thoughts related to each meal and their ED in general [20]. Normally, clinicians review the patient’s diary in the beginning of each treatment session to integrate it in the session [20]. However, effective CBT, including self-monitoring, is challenged by the high dropout rates in ED treatment [15]. These might be explained by patients’ ambivalence toward dismissing the ED [21], patient-reported inadequate amounts of clinician support [2,22], and a lack of patient buy-in to treatment, that is, patients disagreeing with the rationale of the given ED treatment [23]. Additionally, filling in a pen-and-paper diary and bringing it to treatment sessions seems to be outdated, which is supported by patients requesting digital alternatives [24,25].

### Recovery Record: An App for Eating Disorder Management

**Figure 1:**
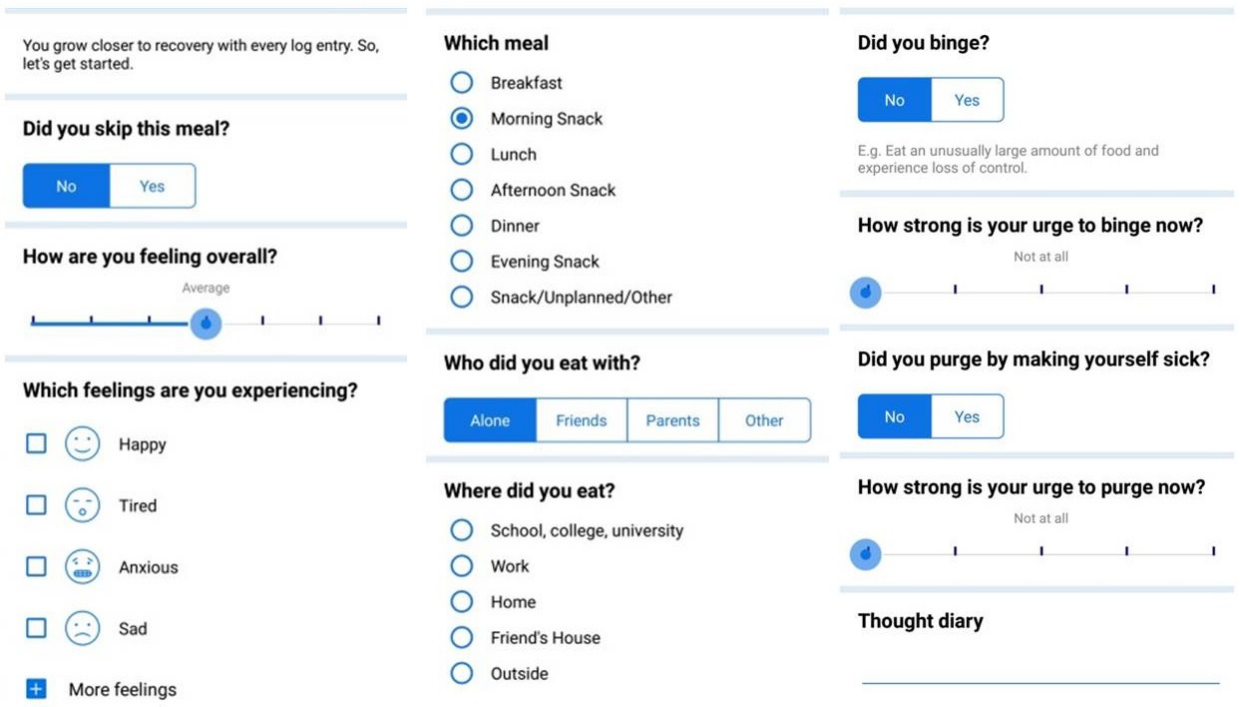
Examples of log questions in Recovery Record. The screenshots are from a Samsung Galaxy S7 (SM-G930F) running Android 7.0.

Recovery Record (RR) is an example of a self-monitoring app for ED management [26]. It works as a self-management tool or as a part of treatment where clinicians employ the clinician interface of the app [26]. Similar to the recognized ED treatment regimens [27,28], RR issues log questions on the user’s meals, behavior, feelings, and thoughts (Figure 1).

It also includes nudging features, that is, meal reminders and affirmations following a meal log, prompting users to self-monitor and eat regularly [26]. RR encompasses other novel features, such as personalized goals and coping strategies, and in-app meal photos intended to increase the user’s incentive to adhere to the app and standard clinical recommendations. RR also contains gamification, that is, game-like principles in a nongame-like setting [29]. In RR, users are rewarded with a piece of a puzzle for every meal log, eventually resulting in a full puzzle linking to a song of their preferred genre [26]. If employed in a treatment program, RR allows for patient-clinician in-app linkage, enabling clinicians to monitor patients’ app data between treatment sessions and providing patients with in-app notifications when their logs have been reviewed [26]. Linking also allows for direct patient-clinician in-app messaging. However, this feature is not permitted in the Danish public health care system because clinicians’ means of digitally contacting patients are restricted [30]. Nevertheless, RR may increase patients’ adherence to ED treatment, including self-monitoring activities, due to its customizable features and digital format, which individuals tend to prefer to pen-and-paper self-monitoring [8,31,32]. In addition, the incidence of EDs is peaking among 15- to 19-year-old individuals [33], which is a tech-savvy age group [3,34]. Finally, patients may prefer in-app meal logging because it is likely to be more discrete than pen-and-paper.

### Apps in Eating Disorder Treatment

Despite their increasing popularity, concerns have also been raised regarding the use of ED treatment apps because it is still uncertain how the quality of psychological treatment is affected when partially disseminated digitally [35]. Additionally, it has been suggested that treatment facilities are prematurely adopting apps out of eagerness to work with novel tools, although their effectiveness and utility remain unclear [36]. However, studies have identified patient-reported benefits of ED management apps; in qualitative studies conducted by Basterfield et al (N=15) and Juarascio et al (N=11), and a usability study with qualitative elements conducted by Nitsch et al (N=9), participants found ED management apps to be convenient and easy to adopt [37]. They also appreciated the option of adjusting the apps to fit their individual needs [24] and found in-app social support from peers and professionals to be helpful in recovery [24,25,37]. However, these findings were based on small samples, in 2 studies, participants without clinically diagnosed EDs were included [25,37], and in the third study, participants did not use but simply discussed a suggested app [24]. Therefore, to ensure an optimal usage of ED management apps facilitating patients’ recovery by engaging them in treatment, we still need knowledge on patients’ experiences and preferences with these apps. Thus, the aim of our study was to explore patients’ experiences using RR (eg, helpfulness, ease of use, and intrusiveness), including their experiences with the app itself and its influence on treatment and everyday life.

## Methods

### Setting

Participants were recruited from a specialized 2-centered ED treatment facility at Aarhus University Hospital receiving around 500 annual referrals. The facility treats patients with moderate to severe EDs in inpatient or outpatient programs. It employs 53 clinicians working in multidisciplinary teams consisting of psychiatrists, psychologists, dietitians, nurses, and physiotherapists, all with a minimum of a bachelor’s degree. The regular treatment of patients with anorexia nervosa depends on the individual patient’s age and situation, for example, living situation and comorbidity. However, typically, it involves family-based treatment or a weekly session altering between group and individual sessions and the latter including family members, if relevant [16]. Regular bulimia nervosa treatment consists of 10 weekly manualized group sessions followed by an additional group or individual treatment, if needed [38]. Nonresponders are offered additional treatment in the day hospital consisting of 3 weekly days of treatment for 16 weeks. In special cases, patients are offered individually tailored programs, for example, in case of severe comorbidity. The facility has been employing a Danish translation of RR since 2014, although not in a standardized way. At the time of data generation, clinicians had received approximately 2 × 2 h of group training on how to use RR in treatment and introduce it to patients, which was carried out by the first author with a clinician-facilitator ratio of about 10:1. Furthermore, clinicians had access to written and illustrated training material and were encouraged to request additional individual training, if needed.

### Interpretive Description

We applied the qualitative methodology of Interpretive Description because it fits the explorative aim of our study and has the objective of informing and improving clinical practice, preferably by discovering “something new” [39]. According to Interpretive Description, field work, including observation sessions, is important to detect the impact of contextual events on the matter being studied. Interpretive Description applies the notion that social influences are formed by people and form people and their actions; on the other hand, it also seeks a nuanced understanding of the individual’s perceptions of the phenomenon of interest [39]. Thus, the methodology draws on selected parts of ethnography, grounded theory, and phenomenology but also differs from the listed traditions by stressing the value of a “research logic,” permitting the researcher to apply and combine the methods needed to fully answer the research question. This flexibility of Interpretive Description is practical when exploring a field, where unexpected findings may occur requiring an adjusted strategy. In Interpretive Description, the validity and relevance of the study are pursued partly by conducting the data generation and analysis simultaneously and partly by keeping a detailed audit trail [39]. The former allows for the early analysis to inform the subsequent data collection that may be adjusted accordingly and vice versa, whereas the latter keeps a track of the preliminary findings and methodological decisions made during the study [39].

### Theoretical Framework

According to Interpretive Description, a theoretical framework may be applied to help set the study in motion [39]. Consequently, because RR is founded upon it, we employed the rationale of CBT focusing on the relationship between physical state, behavior, thoughts, and emotions [20]. We also applied the self-determination theory (SDT) describing how individuals’ actions depend on their personal convictions and the degree to which their psychological needs for competence, autonomy, and relatedness to others are fulfilled [40]. We combined the two because SDT accounts for the individual’s experience of how it is impacted by its context, for example, social setting, to a higher degree than CBT. The theoretical framework influenced the data generation by inspiring the development of the interview and observation guides (Tables 1-2). However, it did not determine the data analysis, in which inductive findings were still allowed [39].

### Data Generation

#### Ethical Considerations

Eligible patients were invited to participate in the study after the initial treatment assessment by the clinician performing the assessment or by the first author. The clinician or the first author provided oral and written information on the study purpose and methods as well as the participants’ right to withdraw at any time without any treatment consequences. After 4-8 weeks, patients who had neither agreed nor declined to participate were reminded of the invitation by their primary clinician. If they agreed to participate, they signed an informed consent form, which was also signed by the legal guardian(s) if the patients were under the age of 18 years. The data material was anonymized and kept confidential. The study was approved by the Danish Data Protection Agency (case ID: 1-16-02-313-15) and conducted according to current legislation [41,42].

**Table 1:** Semistructured interview guide. The guide was adjusted to fit the number of interview participants, who were asked additional follow-up questions as needed, and the order of subjects (in bold) was flexible. The theoretical inspiration column identifies which part of the theoretical framework the questions were inspired by.

Interview guide			Theoretical inspiration
**Patients’ usage of Recovery Record (RR)**			
	Please tell me about the way you use RR on a “normal” day without treatment sessions, for example, when in school or with your friends? Do your friends or family members know about RR?	SDT^a^	
	Which RR features do you use? Why? Are there features you have stopped using? Why?	—	
	Does RR affect your eating and your thoughts and feelings about eating? Is it different for you to log a meal accompanied by eating disorder (ED) symptoms (eg, binging) than to log a meal without ED symptoms? How?	CBT^b^	
	What is it like to log your ED behavior, feelings, and thoughts in RR?	CBT	
	Do you use other apps relating to EDs or diet or calorie counting? Does RR affect how you use these other apps or vice versa? How?	SDT	
**Usage of RR in the patient-clinician collaboration**			
	Please tell me about the way you and your clinician adapted RR to your symptoms, that is, when selecting what to monitor? Did you and your clinician agree on what was important to monitor? Why or why not? If disagreeing, how did you and your clinician proceed?	CBT and SDT	
	How does it feel knowing that your clinician has access to your app data? Do you consider this when logging? Why or why not?	CBT and SDT	
	Are you experiencing that RR affects what you and your clinician discuss during treatment sessions? How?	CBT and SDT	
	How does your clinician use RR in your course of treatment, for example, during sessions? Which features does your clinician apply? How do these features make you feel (eg, notifications informing you that clinicians have reviewed your logs)?	SDT	
	What does it make you feel or think when your clinician has or has not used your logs in RR to prepare your sessions?	CBT	
**Usability of RR**			
	How was the process of downloading, setting up, and beginning to use RR for you? Did you need help from anyone, for example, your friends or clinician?	SDT	
	If you have previously used a pen-and-paper meal diary, how do you like using RR in comparison? What is different? Why is that better or worse?	SDT	
	Do the features, text, images, and menu setup in RR make sense to you? Why or why not? What do you think about them? How do they make you feel?	CBT	
**Potential alterations of RR**			
	In your opinion, how could RR be improved, for example, by additions or alterations?	—	

^a^SDT: Self-determination theory.

^b^CBT: Cognitive-behavioral therapy.

**Table 2:** Observation guide. Field notes were recorded discretely during or immediately after the observations. The theoretical inspiration column shows which part of the theoretical framework the topics were inspired by.

Observation guide		Theoretical inspiration	
**Situation**			
	Who is present (participants)? What is the patient-clinician ratio? Clinicians: Which clinical professions are represented? Patients: How many are present? How long have they been in treatment? What eating disorder diagnosis do they have? Others: Are others present, such as relatives (eg, parents), partners, friends, medical students, or others?		SDT^a^
**Participants**			
	How do participants (patients, clinicians, and others) appear? Mimicry: Which emotions do participants appear to display? Verbal communication: What is the tone of voice and choice of words of participants? Nonverbal communication: What body language are participants using? Do participants have eye contact? Does participants' body language change markedly during the session?		CBT^b^
**Interactions**			
	How do participants interact in relation to Recovery Record (RR)? How, why, and by whom is RR brought up during the treatment session? How is the patient-clinician relationship seemingly affected by RR in the session? Do the participants' mimicry, verbal, and nonverbal communication change when using RR?		SDT
**Activities**			
	Which activities in relation to RR are taking place? Which specific RR activities are taking place? Do activities differ in individual versus group settings? Are specific RR features talked about differently in individual versus group sessions? Who initiates the specific activities relating to RR? How does RR influence any other activities taking place?		CBT and SDT

^a^SDT: Self-determination theory.

^b^CBT: Cognitive-behavioral therapy.

#### Sample Size and Composition

**Figure 2:**
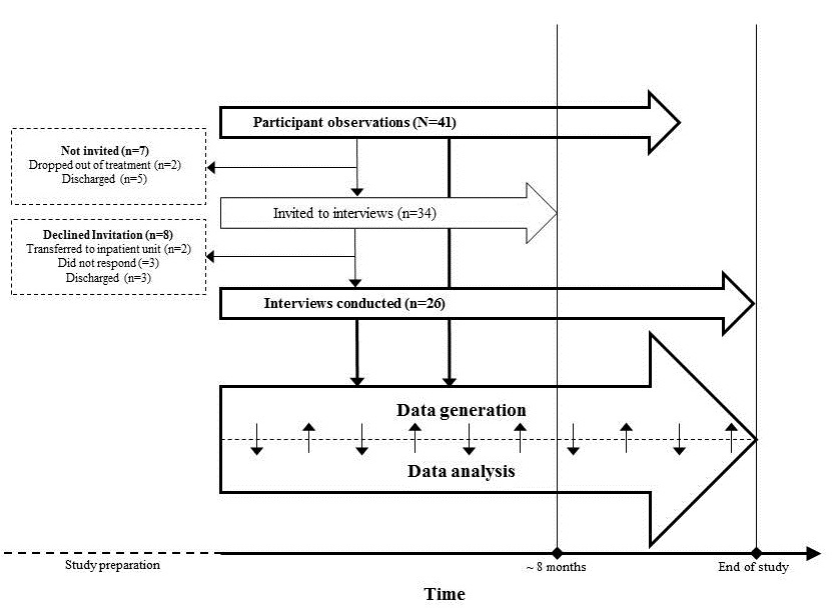
Illustration of participant flow and the concurrent data generation and analysis process. Specified in the boxed are the reasons why some participants were not invited to an interview or declined said invitation.

We recruited patients aged 15 years or older with anorexia or bulimia nervosa. The age limit of 15 years was chosen because younger patients are offered manualized family-based treatment [43] (or other treatment substantially involving the family), which is incompatible with patient self-monitoring [43]. Because patients with BED were not treated at the facility, they were excluded, as were inpatients, because they are continuously monitored by the staff. Patients with psychotic or developmental disorders were also excluded to ensure a participant sample with the cognitive resources needed to perform self-monitoring activities. A total of 41 patients, counting 3 males, were included (Table 3). All 41 were a part of the participant observations, and 34 were invited to an interview, of whom 26 accepted the invitation (Figure 2). Interview participants were sampled purposefully with the aim of obtaining a sample of patients with varying characteristics [39]. In all, 20 participants were interviewed individually, 4 in a focus group, and 2 in a dyadic interview. The dyad took place because 2 additional participants did not show up as planned. Initially, more focus groups were planned with patients attending the same group sessions to capture their perspectives on using RR in group settings, for example, their experiences with clinicians formulating themes across patients using their app data. However, this plan was abandoned because gathering participants outside of treatment sessions was difficult; some were busy with other activities, and others declined because their therapist would be absent. Because ED treatment is often lengthy, we wanted to gather information on the potential changes in patients’ experiences with RR over time. Thus, 5 participants were individually interviewed twice with approximately 6 months in between. These participants were selected purposefully to represent different ED diagnosis, treatment programs, and genders (Table 3). We invited 15 participants for a second interview, but by then, most of the patients had been discharged and no longer wished to participate.

### Data Material

The first author conducted 25 individual interviews (average: 57 min, range: 45-95 min), 1 focus group (94 min), and 1 dyadic interview (83 min), applying a semistructured interview guide to ensure the coverage of subjects relevant to the study aim [39]. To stimulate the discussion in the focus group and dyadic interview, an exercise inspired by Halkier was applied [44]; participants were given printed screenshots of each RR feature and were asked to discuss and comment on the relevance of the features to their treatment. Interviews were conducted at the treatment facility, except for individual interviews of participants who preferred being interviewed at home (n=11).

**Table 3:** Characteristics of participants. Data were collected at the time of the first interview or participant observation session (whichever came first) from self-report questionnaires and medical records.

Variable		Participants (N=41)
Age in years, mean (SD), range		24.0 (5.9), 15-41
Body mass index, mean (SD), range		20.0 (3.5), 15.2-27.6
Previous eating disorder treatment^a^, mean (SD), range		1.3 (1.6), 0-6
Recovery Record usage in months, mean (SD), range		5.5 (6.4), 1-24
**Type of participation, n (%)**		
	Observation sessions	41 (100.0)
	Interviews	26 (63.4)
	Second interview^b^	5 (12.2)
**Age groups, n (%)**		
	15-20	14 (34.1)
	21-25	15 (36.6)
	26-30	8 (19.5)
	≥31	4 (9.8)
**Grouped body mass index, n (%)**		
	15.0-18.4	18 (43.9)
	18.5-19.9	5 (12.2)
	20.0-24.9	12 (29.3)
	≥25.0	6 (14.6)
**Grouped previous eating disorder treatment, n (%)**		
	0	16 (39.0)
	1	11 (26.8)
	2	6 (14.6)
	≥3	7 (17.1)
**Grouped Recovery Record usage in months, n (%)**		
	1-2	14 (34.1)
	3-4	14 (34.1)
	5-6	4 (9.8)
	≥7	9 (22.0)
**Eating disorder diagnosis, n (%)**		
	Bulimia nervosa	19 (46.3)
	Anorexia nervosa restrictive type	18 (43.9)
	Anorexia nervosa binging-purging type	4 (9.8)
**Treatment program, n (%)**		
	Regular bulimia nervosa	15 (36.6)
	Regular anorexia nervosa	11 (26.8)
	Individual	9 (22.0)
	Day hospital	6 (14.6)
**Psychiatric comorbidity^c^, n (%)**		
	None	14 (34.1)
	Depression	12 (29.3)
	Anxiety	7 (17.1)
	Personality disorder	5 (12.2)
	Attention deficit hyperactivity disorder	1 (2.4)
**Daily occupation, n (%)**		
	Student	17 (41.5)
	Working	12 (29.3)
	Sick-leave	8 (19.5)
	Other^d^	4 (9.8)
**Living situation, n (%)**		
	Alone	16 (39.0)
	With parents	13 (31.7)
	With romantic partner	9 (22.0)
	With roommate	3 (7.3)
**Relationship status, n (%)**		
	Single	28 (68.3)
	In a relationship	10 (24.4)
	Married	3 (7.3)

^a^Defined as the number of previous separated courses of eating disorder treatment in public or private facilities.

^b^Characteristics of participants interviewed twice: bulimia nervosa (n=2), anorexia nervosa restrictive type (n=2), anorexia nervosa binging-purging type (n=1); treatment program: regular anorexia nervosa (n=1), regular bulimia nervosa (n=2), individual (n=2); male (n=1).

^c^Some patients had 2 to 3 additional psychiatric diagnoses (n=8).

^d^The term “Other” includes maternity leave and job training arranged by the municipality.

The audio from each interview was digitally recorded and transcribed verbatim by a student worker who had received thorough instructions, for example, on how to mark participants’ tone of voice in the transcripts. The first author evaluated the transcripts by comparing random parts of every fourth manuscript to the corresponding audio recording. Besides minor errors, which were corrected, the transcripts were satisfying.

Because individuals’ verbal statements on their behavior may differ from their actual behavior [39], the interviews were supplemented with participant observations performed by the first author of the individual or group treatment sessions (approximately 160 h) [44]. An observation guide was employed to ensure that the different aspects of patients’ usage of RR and the patient-clinician interaction concerning the app were detected and documented as field notes. Besides exploring participants’ observed behavior in addition to the interview statements, the participant observations inspired the further development and adjustment of the interview guide. The data material was generated in the years 2016-2017.

### Data Analysis

Interview transcripts and field notes were continuously added to and ordered in NVivo 11® [45]. Although the data analysis was a creative and iterative process repeated as new data material was generated, it can, for the sake of clarity, be described as a 4-step procedure [39]. First, we performed a systematic and broad initial coding constantly shifting between the process of coding and taking “a step back” to gain perspective of the data material as a whole. Second, we discarded irrelevant data, namely, data on technical or aesthetic aspects of the app, while retaining the data contributing to our study aim [39]. Third, we described and discussed themes grounded in the remaining data. If we disagreed on or doubted their trustworthiness, we repeated the broad coding to ensure that the themes did indeed derive from the original data material, and that we had not overlooked any contradictory data [39]. Thus, we addressed any inconsistencies both within and between the interview transcripts and field notes. Finally, we described the critically assessed themes [39].

## Results

### Dynamic Patient Experiences

**Figure 3:**
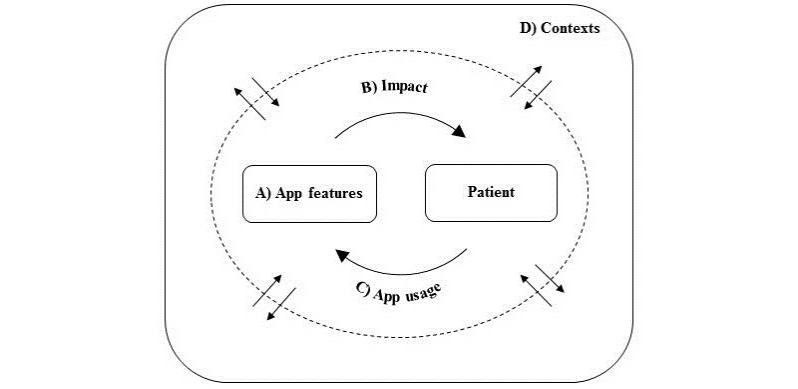
Illustration of results. The figure depicts the patient-app interaction, ie. the interaction between A) the Recovery Record features, B) the impact of these on patients, and C) patients' specific app usage. This patient-app interaction (dotted circle) affected and was affected by D) patients' contexts (outer box), ie. physical location, time of day/week, social settings, treatment program, and the course of time.

We found that the individual patients placed each RR feature along a continuum from supportive to obstructive of their everyday life activities, including school, work, hobbies, social events, and the ED treatment. Thus, patients perceived some features of RR mostly as supportive, for example, affirmations improving their treatment adherence, while experiencing other features primarily as obstructive, for example, meal reminders pinpointing their illness to them when otherwise engaged, for instance, in school work. We found that the various patient experiences with RR depended on A) its features, B) the impact of these features on patients, and C) patients’ app usage, that is, the specific manner in which each patient used RR (Figure 3). This patient-app interaction affected and was affected by patients’ changeable D) contexts, which made their experiences with RR dynamic. Three groups of app features appeared particularly significant to patients’ experiences of RR as mostly supportive or obstructive, that is, features related to logging, nudging, and patient-clinician linkage. Below, we elaborate on the patient-app interaction, but first, we briefly outline the patient-reported contextual factors of importance when using RR.

### Contexts Affecting the Patient-App Interaction

The contexts described by patients as significant to their experiences with RR were physical location (eg, in school), time of day and week (eg, nights and weekends), social setting (eg, with friends), and current treatment program (eg, group treatment). Besides influencing the patient-app interaction, contextual factors affected the patients’ placement of specific RR features along the supportive-obstructive continuum. Using the social setting context as an example, some patients perceived meal reminders as supportive when alone but obstructive when with friends. Moreover, the patient-app interaction could change over time, for example, as patients’ treatment progressed; then, some patients gradually found RR to be more supportive, possibly due to increased treatment buy-in, that is, an elevated acceptance level of treatment guidelines, which may have validated the content of various RR features to patients.

### The Patient-App Interaction

#### Logging: To Log or Not to Log?

Two aspects of the log questions posed by RR were important to patient experiences, namely, their focus and preset format (Table 4). Some patients found it supportive to log because the preset format made them confront the parts of their ED that they would otherwise ignore. This was especially the case after becoming accustomed to the app over time and encouraging continuous logging, as recommended by clinicians.

**Table 4:** Patients’ experiences with the Recovery Record (RR) meal log features. The table summarizes the individual patient-app interaction, that is, the specific features related to meal logs, the impact of these on patients, and patients’ specific app usage supported by interview quotes.

App features and impact			App usage	Interview quotes
**Preset log questions**				
	**Supportive**			
		Confronting the eating disorder	Continuous logging	“If you're struggling with binging and purging, you're kinda forced to log it, 'cause you're asked about it. Previously, it was easier to avoid talking about it if you didn't feel like it.” “I've been able to see a relationship between not eating enough or at the right time of day, and having an urge to binge. So, in that way, logging makes a lot of sense to me.”
	**Obstructive**			
		Pointless if no distress	Avoiding to log	“If I’m doing well at a meal and moving forward, I don’t need it [logging]. If I believe I’ve been eating what I’m supposed to, I don’t see a reason to log.”
		Maintaining the eating disorder	Obsessive logging	“There's this thing in me that wants to keep track of everything. It [logging] was overwhelming and quite intrusive, 'cause it had to be a certain way, and I couldn't change the format. That bothered me a lot, 'cause then I felt like the app controlled me.” “Sometimes, logging gives me ideas. When it [RR] asks me if I've been exercising, I hate answering no. I never replied no in the other [fitness] app. So it gives me an urge to exercise.”
**Focus of log questions**				
	**Supportive**			
		Liberating	From obsessive to constructive logging	“I had to follow a meal plan and log it. And stop weighing myself and counting calories. It was so scary and stressful, but also extremely liberating.”
	**Obstructive**			
		Enslaving	Obsessive logging	“I counted calories using this other app. But then I had to use this [RR] too, so I had to use two apps. It was too much and became strenuous.”

However, other patients found it unnecessary or even obstructive to log if a meal had not caused any significant distress. Then, these patients felt like they were logging simply for the sake of logging, which was considered as pointless and time-consuming. Instead, they would prefer not logging altogether.

Prior to adopting RR, several patients had become habituated to using apps focusing on weight loss or fitness-related content. Some found these apps to be addictive because they made calorie tracking easy, which provided immediate stress relief by reducing their fear of gaining weight. Then, when commencing ED treatment, some patients experienced a similar addiction to logging but this time in RR; although not encouraged in the app, they logged their meals in excessive detail to keep monitoring their calorie consumption. This made some patients log obsessively in RR, which they experienced as obstructive because it partly maintained their ED by making them uphold or resume harmful habits developed when using fitness-related apps. Similarly, the preset log questions, for example, on exercise or purging, could instigate urges to exhibit these behaviors in some patients. Consequently, some succumbed to these urges, whereas others experienced distress due to ongoing deliberations on whether to pursue the urges. To avoid these potentially triggering stimuli, these patients tended to avoid logging. On the other hand, others were relieved by replacing fitness-related apps with RR. Here the log questions focus on emotions and behavior related to each meal and not on calories and weight. Thus, several patients found it liberating to monitor their food intake in a broad sense as opposed to having a strict focus on calories and weight, as is the case in many fitness-related apps. Therefore, some patients therefore experienced logging in RR as supportive because it helped them transform their previously adverse app usage into a recovery-oriented one. However, other patients did not feel ready to solely use RR because the thought of completely abandoning their calorie records in fitness-related apps increased their anxiety levels. Thus, some used RR and a fitness-related app, especially in the initial phases of the ED treatment. This obsessive “double bookkeeping” was experienced as obstructive by patients by being highly time-consuming and enslaving.

##### Nudging: Guidance or Nuisance?

Two nudging features, meal reminders and affirmations, were significant to the patients’ experiences of RR (Table 5). Several patients found the meal reminder feature to be supportive because it provided a structure guiding them to eat and to log the number of meals recommended by clinicians. Other patients experienced meal reminders as nuisances occurring at inconvenient times, for example, when socializing with friends. To some patients, receiving meal reminders was overwhelming because it confronted them with their illness and treatment need when they wanted to focus on other things instead. In addition, others found meal reminders to be ignorant of the core symptom of ED; patients were not simply forgetting to eat but explicitly avoiding it. Finally, meal reminders were experienced as condescending by some patients feeling like they were being treated as incompetent individuals incapable of structuring meals and remembering to eat on their own. Thus, for different reasons, some patients mostly experienced the meal reminder feature as obstructive, making them turn it off or avoid RR altogether.

Receiving an affirmation after a meal log was experienced as supportive by several patients; they felt rewarded for complying with treatment guidelines, which encouraged continued app and treatment adherence. However, other patients found the feature to be negligent of the seriousness of EDs because they considered the feature to be built on the assumption that precomposed messages would speed up their recovery. Moreover, some patients found that the feature addressed them as if they were children, which they experienced as condescending. This was obstructive, especially to patients whose ED had led them to regress in terms of maturity, for example, by moving back in with their parents depending on their support.

##### Linking: Safety or Surveillance?

RR linking features important to patients were data sharing with clinicians, review notifications received when clinicians had checked patients’ logs, and clinicians’ usage of patient-app data in treatment sessions (Table 6). Overall, several patients found the patient-clinician linkage feature to be supportive by making them feel safe; patients expected clinicians to monitor their logs and interfere, if necessary, for example, if they unintentionally neglected any treatment guidelines. Thus, the linkage feature encouraged these patients to log continuously, enabling clinicians to track their treatment progress and interfere if needed. However, the linkage feature caused distress in other patients who felt exposed; not only were their ED symptoms documented in an app but the data were also visible to clinicians. The distress was especially prominent in patients with ED symptoms that they perceived as shameful, for example, binging and purging. Thus, some patients logged their meals leaving out the shameful symptoms, whereas others avoided logging altogether. To these patients, the linkage feature was mostly obstructive due to additional distress.

Several patients found the review notifications to be helpful by reminding them that they were not alone in their recovery efforts; their clinician was “out there.” Thus, by inducing a sense of relatedness in between treatment sessions, the review notifications were supportive to some patients, encouraging them to log continuously and work on their recovery. Yet, the review notifications caused discomfort in other patients who felt being under surveillance, particularly when notifications arrived at unexpected times, for example, on another weekday than expected. This was obstructive to some as it entailed speculations as to why clinicians had reviewed the logs at that specific time. Some patients also had intrusive thoughts about their clinicians’ opinion about their logs, worrying that clinicians were judging or making fun of them when viewing the app data. Subsequently, some patients censored their logs or were discouraged from logging.

**Table 5:** Patients’ experiences with the nudging features of Recovery Record (RR). The table summarizes the individual patient-app interaction, that is, the specific nudging features, the impact of these on patients, and patients’ specific app usage supported by interview quotes.

App features and impact			App usage	Interview quotes
**Meal reminders**				
	**Supportive**			
		Structuring	Continuous logging	“I feel like it [RR] is helping me quite a lot. When I started eating according to the meal plan, it was a good way to make sure that I was actually following the plan. I need that structure in my life in order to eat what I’m supposed to.”
	**Obstructive**			
		Reminder of illness and treatment need	Avoiding to log or turning off feature	“Actually, the app is quite challenging. First, you have to eat. And when you've eaten, you have to log it. So you're reminded that you've eaten. Again. And you just wanna move on.”
		Condescending	Avoiding to log or turning off feature	“It's not like my problem is that I forget to eat, but that I sometimes don't want to.”
**Affirmations**				
	**Supportive**			
		Encouraging and rewarding	Continuous logging	“It’s affirmations like ‘I wanna be kind and loving to myself today’. It’s so basic, but then you think, I haven’t been kind to myself all day. Or maybe the entire week. And the more times you get those hints, the more they stick with you.”
	**Obstructive**			
		Condescending	Avoiding to log	“It seems like it’s supposed to be fun logging all this stuff, but for me, it’s a serious thing that I need to get used to [logging]. It becomes too much fun and games.” “And it’s like ‘here’s a treat for you, since you’ve been good’. And that makes you feel less inclined to recover. It's a bit childish and condescending. When you have this [eating disorder], it's like you’re becoming a kid again, 'cause you can’t eat on your own. That's reinforced by the app treating you like a child.”

Some patients found it invasive, yet helpful, when clinicians explicitly used the app data in sessions, for example, by highlighting patients’ attempts to resist the urges of ED. Patients felt that they were taken seriously when they got the impression that their clinician had thoroughly prepared the session using the app data, which encouraged them to keep logging. Nonetheless, others found it obstructive to know that clinicians could use their app data in session; it made them worry about their clinician’s judgment prior to each session. Consequently, some patients excluded information that they expected their clinician to disapprove. Other patients would prefer if clinicians only viewed their logs during sessions as opposed to before, allowing them to have a dialogue about and explain what was logged. Most patients had experienced clinicians not employing any app data in session, which was disappointing because they had made an effort logging, partly with the aim of the logs being commented on. Some patients felt like their clinicians were neglecting their professional responsibilities when seemingly not reviewing patient logs and utilizing them in session. Consequently, some patients lost trust in their clinicians. Thus, it was primarily obstructive when clinicians did not incorporate the app data in treatment sessions.

**Table 6:** Patients’ experiences with the patient-clinician linkage feature in Recovery Record (RR). The table summarizes the individual patient-app interaction, that is, the specific linkage features, the impact of these on patients, and patients’ specific app usage supported by interview quotes.

App features and impact			App usage	Interview quotes
**Data sharing**				
	**Supportive**			
		Feeling safe	Continuous logging	“It provides some kind of security knowing that someone is keeping an eye on me. It makes me feel safer.”
	**Obstructive**			
		Feeling exposed	Avoiding to log or Logging with clinicians in mind	“I wasn't always honest about it [exercising]. Often, I'd just log 'no'. I was embarrassed to admit it to my clinician.” “I cheat quite a lot. Those days when I don't log, it's because I feel bad about not eating what I was supposed to.”
**Review notifications**				
	**Supportive**			
		Feelings of relatedness	Continuous logging	“I like them [review notifications]. It's part of treatment. It reminds me that I'm doing this [eating disorder treatment]. And they [clinicians] are here to help.”
	**Obstructive**			
		Feeling under surveillance	Avoiding to log or Logging with clinicians in mind	“It makes me wonder why they've been looking at my logs at that specific time. If it's in the middle of the week and my appointment isn't until a week later, then I start wondering why they're looking.” “It makes me worry. Like, are they laughing at me? Or judging me. It makes my heartbeat rise.”
**Clinicians using logs in sessions**				
	**Supportive**			
		Encouraging	Continuous logging	“They'll check if you've lost or gained weight [using a scale]. And then they confront you saying look at your app data. You haven't been eating like you should. It's kinda intrusive, but also really helpful getting that push. You need it.” “It makes me so proud when I succeed and they [clinicians and other patients] see it.”
	**Obstructive**			
		Concerned about confrontation	Logging with clinicians in mind	“It was kinda like she had to control that I had been doing things correctly. It made me wonder what would happen if I had done something wrong, or hadn't been doing well enough.” “I'd rather she'd just look, when we meet face-to-face, so I can say something.”
**Clinicians not using logs in sessions**				
	**Obstructive**			
		Feeling neglected	Avoiding to log	“She said she'd go through my logs before our sessions, but I feel like that didn't actually happen. There were no consequences. If I'd logged something specific, she didn't ask about it, although I was expecting it. Then it's like it doesn't really matter what I do.” “If they wanna use it [RR], it should be obvious to them that they should comment on my logs. If they don't, I don't mention it. I don't wanna seem needy.”

## Discussion

### Minimally Disruptive Medicine

In this study, we found that patients’ experiences with RR were dynamic and depended on the individual patient-app interaction. Some patients primarily experienced the RR features as supportive of their everyday life activities, including the ED treatment, for example, by supporting a regular eating pattern and by inducing a sense of relatedness to clinicians. In contrast, other patients mostly perceived the app or its features as obstructive of day-to-day life, for example, when feeling being under surveillance or when transferring obsessive logging behavior from other apps to RR. Thus, our findings add to the field by highlighting the complex diversity of patient experiences using an app such as RR and with that the importance of adjusting technological treatment tools to fit the individual patient.

The concept of minimally disruptive medicine may explain part of our findings; it aims at offering effective treatment that also fits the individual patient’s preferences and daily life [46]. In minimally disruptive medicine, managing the workload, that is, tasks and responsibilities, associated with long-term treatment requires substantial patient capacities, for example, individual and contextual resources [46]. If the treatment workload exceeds the patient’s capacities, they feel burdened and may reduce their adherence to treatment, thereby decreasing its effectiveness [47]. Thus, clinicians and patients need to collaborate to reach a patient workload-capacity balance [46]. Transferred to our study, some patients might have experienced RR mostly as obstructive of daily life because the workload accompanying the app exceeded their overall capacities or conflicted with their preferences. Thus, patients and clinicians need to assess the various app features together, while taking patients’ day-to-day life activities, preferences, treatment needs, and capacities into account. Consequently, the supportive app features may be applied further, whereas the obstructive ones may be avoided. However, this patient-clinician collaboration may be challenging because tools assessing patient capacities and preferences are lacking [47]. Furthermore, the patients’ perception of treatment workload may depend on their abilities to counteract the ED pathology in general; in ED treatment, a common challenge is the egosyntonic nature of some ED symptoms and patients’ ambivalence toward some treatment activities, for example, weight gain [48]. Finally, the clinicians’ reception of the training on how to use RR and with that their specific RR usage [49] may also influence the patients’ experiences of treatment workload. Thus, minimally disruptive medicine might be especially complicated to apply in ED treatment. Still, we recommend clinicians to focus on how self-monitoring apps may best fit the individual patient’s preferences and treatment needs.

Clinical practice is in need of explicit guidelines on the usage of apps in ED treatment [49]. Although the specific content and design of such guidelines require more research, our study outlines possible recommendations. Overall, patients and clinicians need to explicitly discuss how to apply a specific app in treatment and the patient’s everyday life. Specifically, we recommend a discussion of (1) the degree of helpfulness of app features to determine which should be applied and how, (2) the parties’ expectations to one another regarding the usage of app data in and outside treatment sessions, including who is responsible of introducing the data in session, (3) specific issues related to possibly harmful, obsessive logging, and (4) each patient’s specific reasons for potentially not logging. We recommend these points to be discussed continuously during the course of treatment because the individual patient’s needs and preferences might change over time. Finally, we encourage app developers to ensure that the apps are flexible, allowing for specific features to be easily selected or deselected in accordance with the preferences and treatment needs of individual patients. Besides further research on the content of clinical guidelines, our findings pinpoint the need for studies investigating the treatment effect of RR and similar apps, the positive and negative effects of specific app features on patient sub groups, and predictors of app usage, for example, patient characteristics.

### Strengths and Limitations

To our knowledge, our study is the first to explore in-depth the patient experiences using an ED treatment app in a naturalistic ED treatment setting, which is an important step toward filling the gap in the literature. By generating data in individual and group settings, we have covered the aspects affecting the patient-app interaction on both these levels. However, the group dynamics of relevance when using RR in group treatment might have been elaborated on further, if more group interviews had been feasible. It is also important to consider the potential impact of the cultural setting on the study participants and with that the findings; in Denmark, public health care is free of charge, which might reduce the influence of social and financial resources on treatment experiences, as found in other countries [50]. The dismissal of the in-app messaging feature could also have biased our results toward more negative patient experiences with RR, for example, by limiting their feelings of relatedness. Moreover, our findings might have been skewed by the fact that some participants declined or did not respond when invited to an interview. Nevertheless, because the remaining participant sample is rather diverse in terms of patient characteristics, we expect having portrayed significant experiences of various patient groups. Although our novel approach of interviewing participants twice provided some understanding of the perspectives on using RR over time, more than 5 participants would likely to have benefited our study. Finally, there are several RR features that we have not dealt with in this paper because patients did not point them out as significant. However, rather than patients not finding these features as important, their disregard of some features might be associated with the nonstandardized clinician app usage at the ED treatment facility, or a lack of clinician training or technological abilities in some clinicians. Thus, ED treatment facilities and clinicians should keep in mind that the remaining app features might still benefit patients if applied appropriately.

### Conclusions

Patients’ experiences with RR in ED treatment varied and depended on their individual app interaction and contextual factors. Some patients experienced RR mostly as supportive of their everyday life and ED treatment, whereas others experienced the app and its specific features primarily as obstructive. Thus, when applying apps in ED treatment, we recommend that patients and clinicians collaborate to clarify how the app in question best fits the individual patient’s capacities, preferences, and treatment needs. Similarly, we encourage app developers to build flexible apps that may easily be adjusted to fit individual patient’s preferences and treatment needs.

## Abbreviations

ED: eating disorder
BED: binge eating disorder
CBT: cognitive-behavioral therapy
SDT: self-determination theory
RR: Recovery Record
